# Effect of Gamma Radiation on Cytogenetic and Growth Endpoints of *Allium cepa* Seedlings in Long-Term Experiments

**DOI:** 10.1134/S1607672922020028

**Published:** 2022-05-10

**Authors:** A. Ya. Bolsunovsky, E. A. Trofimova, A. V. Zueva, D. V. Dementyev, M. V. Petrichenkov

**Affiliations:** 1 Institute of Biophysics, Federal Research Center “Krasnoyarsk Science Center SB RAS”, Siberian Branch, Russian Academy of Sciences, Krasnoyarsk, Russia; 2 Budker Institute of Nuclear Physics, Siberian Branch, Russian Academy of Sciences, Novosibirsk, Russia

**Keywords:** Allium-test, seedlings, gamma irradiation, chromosome aberrations, recovery time, inhibition

## Abstract

The effect of γ-radiation on the growth and cytogenetic endpoints of *Allium cepa* L. seedlings in a long period after irradiation in absorbed doses from 1.0 to 10.7 Gy were examined. The chromosome aberration rate was most sensitive to the radiation: it increased immediately after exposure at all doses. In the recovery period (up to 216 h) after irradiation, the chromosome aberration frequency naturally decreased but at the end of experiment in maximum doses remained above the control values. The impact of the initial level of chromosome aberrations on the inhibition of onion roots growth in the long terms after irradiation is discussed.

Nuclear weapons testing and the activities of nuclear fuel cycle enterprises have led to the release of a significant amount of technogenic radionuclides into the environment. Hydrobionts of the Yenisei River experience an additional dose load due to technogenic radionuclides, including those in the form of radioactive microparticles, as a result of long-term industrial discharges of the Mining and Chemical Combine of the State Corporation Rosatom into the river [1–4]. Previously, we performed laboratory experiments with various bioassays to simulate the effect of γ-radiation of radioactive particles and showed the high sensitivity of bioassays to γ-radiation [3, 5–7].

In toxicological experiments, we used the onion biotest (*Allium*-test) [6, 7], which has shown itself well in assessing the toxicity of environmental samples [8–11]. For onion seedlings, an increase in the frequency of cytogenetic disorders in cells was found at all doses of γ-irradiation for 24 h, including low doses [6]. At the maximum absorbed dose of 13 Gy, chromosome aberrations were observed in the majority of onion cells at the anaphase and telophase stages of mitosis, and the number of cells with multiple disorders increased. Previously, it was noted that DNA damage in the cells of living organisms can lead to termination of their growth, as well as to long-term consequences [12]. However, in our experiments, at a high level of chromosome aberrations, no effect of radiation on root growth was revealed [6]. The absence of inhibition of growth processes can be explained by both relatively low doses of γ-irradiation and the short duration of our experiments (24 h) [6]. A number of authors described the inhibition of plant growth parameters after irradiation; however, they used high doses [12–15]. For example, in the review by Gudkov et al. [12], plant growth inhibition was observed at radiation doses from 5 to 50 Gy and higher, in the study by Liu et al. [15] the growth of wheat roots was inhibited at doses above 30 Gy. Many authors do not suggest the mechanisms of inhibition of the growth of irradiated plants. In the study by Zaka et al. [16], when pea seedlings were irradiated at doses up to 10 Gy, inhibitory effects on plant growth were noted, which were recorded after several generations. According to the authors, the inhibition of plant growth in the second generation can be explained by genomic instability induced by irradiation [16]. However, the authors provide data on the levels of cytogenetic disorders only at the initial moment of plant growth (0 and 20 h after irradiation). For an objective assessment of the long-term effects of γ-irradiation in experiments, long-term observations of not only the growth but also the cytogenetic parameters of onion seedlings after irradiation are required.

The aim of this work is to evaluate the effect of γ-radiation on cytogenetic and growth parameters of onion *Allium cepa* L. seedlings in the long terms after irradiation.

The seeds of onion (*Allium cepa* L.) (2*n* = 16) of the Stuttgarter Risen variety were used in the experiment. Seeds were stored at 4°C in commercial packs until they were used in experiments. Seeds were germinated in plastic containers on filter paper moistened with distilled water in the dark at room temperature. For irradiation, seedlings 2–3 mm long were used (11 to 130 pieces, depending on the experiment, for each dose level). Irradiation was performed for 24 h with a point source placed in a steel capsule and containing ^137^Cs (activity 14 GBq at the time of experiments) at the Budker Institute of Nuclear Physics, Siberian Branch, Russian Academy of Sciences (Novosibirsk). The dose rate values were determined by the distance of seedlings from the source and were verified by direct measurements with dosimeters (DKG-02U, NPP Doza, Russia and DKS-AT1123, Atomtech, Belarus). Two experiments were performed: in Experiment No. 1, onion seedlings after irradiation for 24 h were additionally grown for 24 h without irradiation; in Experiment No. 2, onion seedlings after irradiation for 24 h were additionally grown without irradiation for 24, 72, 120, and 216 h. In Experiment No. 1, plants were irradiated at three dose levels, where the absorbed dose was 1.0, 2.6, and 5.2 Gy. In Experiment No. 2, plants were irradiated at four dose levels, where the absorbed dose was 1.0, 2.6, 5.2, and 10.7 Gy. The selection of   radiation doses was based on the previously obtained dose dependence for cytogenetic disorders of *Allium cepa* [6]. The absorbed dose for control plants was 10^–4^ Gy.

The length of roots and the proportion of abnormal cells with various types of disorders at the ana-telophase stage of mitosis were used as indicators of radiation damage. Cytogenetic parameters were determined using a light microscope (Olympus CX31) on squashed samples prepared by the standard method [6]. To account for aberrant cells, 11 to 26 samples were viewed for each exposure level. The following types of aberrations were noted: fragments, bridges, wandering chromosomes, multiple disorders (the presence of several disorders of different types in the cell), etc. The frequency of chromosome aberrations was calculated as the ratio of the number of cells with aberrations to the total number of ana-telophase cells.

Statistical analysis and visualization of the obtained results were performed using Microsoft Office Excel 2013 and PAST 3.23 software. To compare the irradiated samples with the control, as well as to identify differences between the samples fixed immediately and later, the Kruskal–Wallis test with pairwise comparison of groups using Dunn’s test with Bonferroni correction was used.The figures show the mean values and the standard errors or the mean.

In Experiment No. 1, after 24 h of irradiation, an increase in the frequency of chromosome aberrations (almost 100%) in the cells of onion seedlings compared with control was observed (Fig. 1a). This effect was observed at all doses used. However, no significant inhibition of root growth during irradiation was observed (Fig. 1b), despite a slight decrease in the average root length at the maximum dose of 5.2 Gy to 8.7 mm compared with the root length in the control (11 mm).

**Fig. 1.**
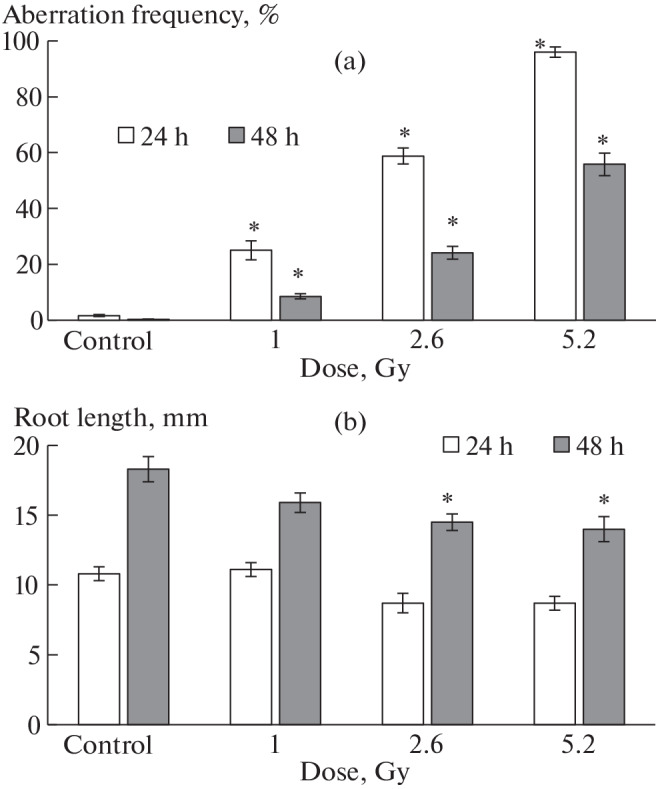
Effect of different γ-radiation doses in Experiment No. 1 on chromosome aberration frequency (a) in cells of onion seedling and on root length (b) immediately after irradiation (24 h) and after growing plants without irradiation (48 h). * Significant differences from control (*p* < 0.05).

During the recovery period of 24 h, a nearly twofold decrease in the frequency of chromosome aberrations at all doses was observed. For example, at the maximum dose of 5.2 Gy, the frequency of aberrations decreased from 96 ± 2 to 56 ± 4%; at a dose of 2.6 Gy, from 59 ± 3 to 24 ± 2% (Fig. 1a). The range of chromosome aberrations during the recovery period did not change compared to the irradiation period: multiple disorders and fragments continued to dominate. After irradiation, the decrease in the frequency of chromosome aberrations was mainly due to a decrease in the proportion of multiple disorders.

Despite the decrease in the level of cytogenetic disorders in the 24-h recovery period, root growth inhibition to 14 mm was recorded at doses of 2.6 and 5.2 Gy (Fig. 1b) compared with the average root length in the control (18.3 mm). It is likely that the effect of γ-irradiation on growth processes is of an inertial nature compared to cytogenetic disturbances.

In Experiment No. 2, in contrast to Experiment No. 1, the maximum level of the dose of γ-irradiation increased to 10.7 Gy and the duration of the experiment increased from 48 to 240 h, i.e., the recovery period of plants after irradiation significantly increased. Despite the twofold increase in the maximum irradiation dose, in Experiment No. 2, as well as in Experiment No. 1, after 24 h of irradiation, an increase in the frequency of chromosome aberrations with increasing dose and the absence of inhibition of root growth were noted at all doses used (Figs. 2a, 2b).

**Fig. 2.**
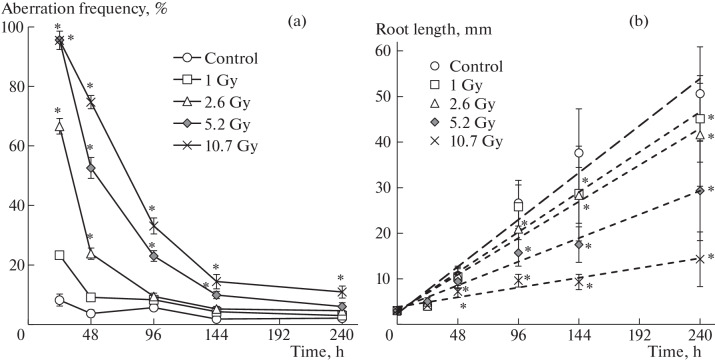
Effect of different γ-radiation doses in Experiment No. 2 on chromosome aberration frequency (a) in cells of onion seedling and on root length (b) immediately after irradiation (24 h) and after growing plants without irradiation. Dashed lines—linear approximations (Table 1). *Significant differences from control (*p* < 0.05).

At a recovery period of 24 h, as in Experiment No. 1, a decrease in the frequency of chromosome aberrations was also observed at all doses. However, the degree of decrease differed at different radiation doses: at the maximum dose of 10.7 Gy, the frequency of aberrations decreased insignificantly from 96 ± 3% to 75 ± 2%; at a dose of 5.2 Gy, the aberration frequency decreased from 96 ± 1% to 53 ± 4%; at a dose of 2.6 Gy, it decreased from 67 ± 3% to 24 ± 2% (Fig. 2a). At a longer recovery period (more than 24 h), the frequency of chromosome aberrations continued to decrease; however, even for 144–240 h of experiment at doses of 5.2 and 10.7 Gy, the level of cytogenetic disorders in new generations of cells was higher than in the control (Fig. 2a). The cell cycle of onion meristematic cells takes about 23 h, of which 4 h are mitosis [17]. Therefore, in our experiment, no complete repair of damaged chromosomes in cells was observed 6–10 generations after irradiation. It should be noted that the range of chromosome aberrations in the initial recovery period up to 96 h of the experiment did not change compared to the irradiation period: multiple disorders and fragments continued to dominate. During this recovery period, the decrease in the frequency of chromosome aberrations was mainly due to a decrease in the proportion of multiple disorders. Starting from the 144th hour of the experiment, the proportion of disorders such as bridges in the spectrum of chromosomal aberrations increased; they could dominate in the total frequency at the maximum radiation doses.

In Experiment No. 2, similarly to the data of Experiment No. 1, in the 24-h recovery period after the end of irradiation, root growth inhibition to 7.2 mm was recorded at maximum doses of 5.2 and 10.7 Gy (Fig. 2b) compared to the average root length in the control (10.7 mm). With an increase in the recovery time (more than 24 h), root growth inhibition was observed not only at the maximum irradiation doses but also at other doses: inhibition at a dose of 2.6 Gy began at 96 h, and at a dose of 1 Gy it began at 144th hour of the experiment. The linear equation (*l = l*_0_ *+* ${v}t$) with high coefficients of determination (*R*^2^ = 0.93–0.99) is well suited to describe root growth under irradiation and in the control (Fig. 2b, Table 1).

**Table 1.** Coefficients of the equations of linear growth of *l = l*_0_ *+* ${v}t$ at different radiation doses

	${v}$	*l*_0_	*R* ^2^
Control	0.215	2.4	0.97
1 Gy	0.183	2.6	0.97
2.6 Gy	0.167	2.9	0.99
5.2 Gy	0.108	3.4	0.98
10.7 Gy	0.045	3.8	0.93

The linear model characterizes the vegetative growth of irradiated and control onion seedlings as a process lasting in time at a constant rate (the model parameter is ${v}$). As follows from the data shown in Table 1, the root growth rate at the maximum doses of 5.2 and 10.7 Gy is 2–5 times lower than the root growth rate in the control. It should be noted that the root growth rates under irradiation with doses of 1 and 2.6 Gy did not differ from each other but differed from the control by no more than 20%. From the data in Fig. 2b, it follows that the differences in the root growth rate for different irradiation doses and in the control formed in the initial period of the experiment, 24 or 48 h. What happened to the roots during this initial period (24–48 h)? During this period, we recorded the maximum values of chromosomal aberrations (Figs. 1a and 2a) in cells (67–96% at 24 h of the experiment and 24–75% at 48 h of the experiment (the first recovery period)) at irradiation doses from 2.6 to 10.7 Gy. Then, probably, there is a threshold level of chromosomal disorders, which leads to subsequent root growth inhibition. From the experimental data obtained by us, we can assume that the value of the possible threshold level does not exceed 24 ± 2%. At such a minimum frequency of chromosome aberrations, root growth inhibition was observed in Experiment No. 2 at an irradiation dose of 1 Gy (Fig. 2b) after 144 h of the experiment (120 h after irradiation). In Experiment No. 1, root growth inhibition was observed after 48 h of the experiment at a dose of 2.6 Gy and a frequency range of chromosomal disorders of 24–59% (Fig. 1). This additionally indicates a threshold level not exceeding 24 ± 2%.

In reviews [12, 13], numerous cases of inhibition and stimulation of growth parameters of plants after irradiation with high doses were noted. In the study by Amjad and Anjum irradiated at doses of 10, 20, 80, and 100 krad were more sensitive to radiation than sprouts. In the study by Liu et al. [15], wheat seeds were irradiated with heavy carbon ions, and the range of doses used was 10 to 200 Gy. Liu et al. [15] noted the inhibition of grown wheat roots after 3 days in the case of irradiation with doses above 30 Gy and after 7 days in the case of irradiation with doses above 60 Gy. In our experiments (Figs. 1, 2), onion root growth inhibition was recorded several days after γ-irradiation with very low doses, starting from 1.0 Gy. Vaijapurkar et al. [9] observed changes in the growth parameters of onion bulbs (*Allium cepa*) 5 days after irradiation with high doses (more than 5 Gy), while the difference in cytogenetic parameters (mitotic index and number of micronuclei) from the control appeared 2 days later at lower doses of 2–4 Gy. In some studies, the observed effects of inhibition of plant growth parameters after irradiation were explained by cytogenetic disorders and DNA damage [12, 16]. It is known that cytogenetic disorders are one of the most sensitive parameters of γ-irradiation and occur almost immediately after irradiation. Given this fact, the growth inhibition of irradiated pea seedlings (doses up to 10 Gy) in the second generation is explained in [16] by genomic instability of plants induced by irradiation. However, Zaka et al. [16] do not provide data on the levels of cytogenetic disorders for plant growth inhibition.

In the review [12], it is stated that lesions of DNA molecules are one of the main causes of death of living organisms after irradiation. DNA single- and double-strand breaks formed as a result of irradiation can be repaired; however, as a result of repair, errors occur that can lead to changes in genome regulation. In our previous work [18], we assessed damage to the nuclear DNA of onion seedlings using the comet assay and showed an increase in DNA damage in the range of γ-irradiation doses from 0.02 to 5 Gy compared with the control. For the dose dependence of the DNA damage parameter, a non-linear character was noted: a linear section in the region of low doses and a dose-independent plateau in the dose range from 1 to 5 Gy [18]. Possibly, at low irradiation doses, cells can effectively repair emerging DNA breaks. In this case, the presence of unrepaired DNA double-strand breaks can trigger cell death with an increase in the irradiation dose [12]. Therefore, the threshold level of accumulated chromosome aberrations in onion cells, proposed above to explain the root growth inhibition, is obviously determined by the degree of DNA damage. Changes in the cell genome as a result of accumulated chromosome aberrations (chromosomal instability) after irradiation subsequently lead to inhibition of root growth processes. As follows from our data, chromosome damage after the end of irradiation is recorded in cells even after 6–10 generations, and root growth inhibition is observed (Fig. 2).

Thus, the onion bioassay based on *Allium cepa* seedlings, which was used by us, showed for the first time an increase in both the overall frequency of cell chromosome aberrations in anaphase and telophase and root growth inhibition after γ-irradiation in the dose range from 1.0 to 10.7 Gy. The maximum response of the parameters of irradiated plants was recorded at different times of the recovery period: the maximum frequency of chromosome aberrations was observed in the first 24 h after irradiation, and maximum inhibition of root length was observed 216 h after irradiation. Recording chromosome damage in onion cells (for a long time after cessation of irradiation) allows us to consider chromosomal instability as the main cause of root growth inhibition. The data obtained make it possible to use the *Allium cepa* bioassay to assess the long-term effects of γ-radiation during the life cycle of a plant or in subsequent generations.
